# Prebiopsy Steroids and Diagnostic Yield in Patients With Diffuse Large B-Cell Lymphoma

**DOI:** 10.1001/jamanetworkopen.2025.48617

**Published:** 2025-12-11

**Authors:** Sathwik Madireddy, Sovijja Pou, Kaavya Mandi, Kelly Pan, Alex Friefeld, Huang He Ding, Christina Raker, Ross Terada, Charles J. Milrod, Ari Pelcovits

**Affiliations:** 1 Department of Internal Medicine, Brown University Health, Providence, Rhode Island; 2 Division of Hematology/Oncology, Brown University Health, Providence, Rhode Island

## Abstract

**Importance:**

Corticosteroids are frequently used to manage symptoms in patients with diffuse large B-cell lymphoma (DLBCL), but longstanding clinical caution suggests that prebiopsy administration may reduce diagnostic accuracy. The association between steroids and biopsy outcomes in patients with systemic DLBCL remains unexamined.

**Objective:**

To determine whether corticosteroid use before biopsy is associated with diagnostic yield in patients with newly diagnosed DLBCL.

**Design, Setting, and Participants:**

Retrospective case series of adult patients newly diagnosed with DLBCL at a single institution tertiary academic medical system affiliated with Brown University between 2015 and 2024. Patients were identified through institutional hematology and pathology databases. Patients with relapsed or refractory DLBCL or primary central nervous system lymphoma were excluded. Logistic regression was used to assess the association between steroid exposure (dose, duration, and withdrawal) and diagnostic yield.

**Exposure:**

Corticosteroid administration within 30 days before tissue biopsy.

**Main Outcomes and Measures:**

Diagnostic yield of initial biopsy for DLBCL, repeat biopsy rates, and time from first negative biopsy to chemotherapy initiation.

**Results:**

Of 320 patients (mean [SD] age, 68 [14] years and 164 [51%] female), 48 (15%) received corticosteroids prebiopsy. Diagnostic yield was 83% (40 of 48 patients) in the steroid group and 81% (221 of 272 patients) in the nonsteroid group, with no statistically significant difference between the groups (prevalence ratio [PR], 1.03; 95% CI, 0.89-1.18). Among patients with negative first biopsies, time to treatment initiation did not differ significantly between groups. No significant associations were observed between diagnostic yield and total steroid dose, duration, or days off steroids before biopsy. Biopsy type was significantly associated with diagnostic yield. Compared with excisional and incisional biopsies, core needle and fine needle aspiration were 12% (PR, 0.88; 95% CI, 0.81-0.96; *P* = .01) and 69% (OR, 0.31; 95% CI, 0.18-0.63; *P* < .001) less likely to be diagnostic, respectively.

**Conclusions and Relevance:**

In this case series of 320 patients with DLBCL, prebiopsy corticosteroid administration was not associated with reduced diagnostic yield or delay in treatment in patients with newly diagnosed DLBCL. Biopsy technique was most associated with diagnostic success. These findings support a clinically pragmatic approach to steroid use during lymphoma evaluation.

## Key Points

**Question:**

Is corticosteroid administration before biopsy associated with diagnostic yield in adult patients with newly diagnosed diffuse large B-cell lymphoma (DLBCL)?

**Findings:**

In this cohort study of 320 patients with DLBCL, diagnostic yield did not significantly differ between those who received corticosteroids before biopsy and those who did not. There was no statistically significant association between diagnostic yield and steroid dose, duration, type, or withdrawal status.

**Meaning:**

These findings suggest that corticosteroids may be safely administered before biopsy in symptomatic patients with DLBCL without compromising diagnostic accuracy or delaying treatment initiation.

## Teaser

This cohort study evaluates whether corticosteroid use before biopsy is associated with the diagnostic yield in patients with newly diagnosed diffuse large B-cell lymphoma.

## Introduction

Diffuse large B-cell lymphoma (DLBCL) is an aggressive malignant neoplasm associated with substantial early morbidity, including systemic symptoms and organ dysfunction.^1,2,3,4^ Furthermore, due to its rapid progression, DLBCL necessitates timely diagnosis and treatment, with a direct association between diagnosis to treatment interval and long-term survival.^5^ During this initial diagnostic period, patients often require corticosteroid therapy to prevent organ compromise and relieve symptoms. Diagnostic work up and therapeutic interventions, however, are frequently complicated by the concern that corticosteroid administration before biopsy may reduce diagnostic accuracy.

Tissue biopsy remains essential for the histopathologic and immunophenotypic evaluation of DLBCL, as treatment decisions hinge on diagnostic accuracy. Steroids induce cytolysis of malignant B cells, resulting in apoptosis and tumor necrosis, and are therefore hypothesized to compromise diagnostic yield.^6,7,8^ Steroid-induced B-cell lysis can lead to increased infiltration by reactive macrophages and lymphocytes, potentially obscuring diagnostic features.^8,9,10^ In addition, steroids may alter cluster of differentiation 20 (CD20) immunoreactivity—a key diagnostic marker—leading to patchy or atypical staining patterns.^9,10^ Radiologically, this effect has been observed as rapid tumor involution on imaging, earning the descriptor ghost tumor.^11^ Nonetheless, more recent evidence suggests that a steroid administration before baseline positron emission tomography or computed tomography does not diminish diagnostic yield in DLBCL.^12^

The literature evaluating the impact of prebiopsy steroids on diagnostic yield in lymphoma is sparse and limited in scope. Case series have examined rare lymphoma subtypes, such as ocular lymphoma and T-cell or histiocyte-rich large B-cell lymphoma, providing limited data.^13,14^ A pediatric study on mediastinal lymphoma found that steroid exposure could impair pathologic diagnosis and delay definitive treatment, though it also reduced the risk of cardiorespiratory morbidity in high-risk patients.^15^ The most extensive body of work concerns primary central nervous system lymphoma (PCNSL), where steroids are often administered early due to cerebral edema and neurologic deficits.^9,16,17,18,19^ Findings across these studies are mixed, with newer evidence indicating that corticosteroids may not decrease the likelihood of a diagnostic biopsy.

To date, the impact of prebiopsy corticosteroids on diagnostic yield in adult patients with systemic (non–central nervous system [CNS]) DLBCL remains unexamined. The primary objective of this study was to evaluate the association between corticosteroid use before biopsy and diagnostic yield in patients with DLBCL.

## Methods

This study was reviewed and approved by the Brown University Rhode Island Hospital institutional review board, and a waiver of informed consent was granted due to its retrospective design. This report follows the Strengthening the Reporting of Observational Studies in Epidemiology (STROBE) reporting guidelines for case series studies. Specifically, the STROBE items were addressed as follows: the study design and setting are described, participant selection and eligibility criteria are detailed, variables and data sources are clearly defined, statistical methods including handling of missing data are outlined, and results are reported with descriptive data and outcome measures.

### Study Design and Population

We conducted a retrospective medical record review of patients diagnosed with DLBCL at our institution. Patients were identified through cross-referencing institutional databases from the Hematology and Pathology departments across 3 hospitals affiliated with Brown University to ensure data completeness and minimize participant loss. All included cases had initial diagnostic evaluations performed between 2015 and 2024.

### Data Collection

Data were extracted from electronic medical records and included demographic information (age at diagnosis and gender), lymphoma characteristics, corticosteroid exposure within 30 days before biopsy (dose, duration, tapering status, and steroid-free interval if applicable), and biopsy characteristics. Different steroid formulations were converted to prednisone equivalent doses. The lymphoma characteristics included lactate dehydrogenase (LDH) levels, disease stage (according to the Lugano classification), International Prognostic Index (IPI), and double-hit status (a high-risk feature characterized by *MYC* and *BCL2* and/or *BCL6* rearrangements). Biopsy data encompassed type (eg, core needle biopsy, fine needle aspiration, or surgical excision), initial diagnostic yield for DLBCL, and whether additional biopsy procedures were required due to nondiagnostic findings.

Biopsy diagnostic classification was determined based on pathology reports. A biopsy was defined as diagnostic if it demonstrated a high probability of DLBCL based on histologic and/or molecular findings. A nondiagnostic biopsy was characterized by diagnostic uncertainty or lack of definitive evidence for DLBCL. Given the current reliance on integrated diagnostic modalities, molecular data were considered in the classification of biopsy outcomes. Cases of DLBCL that had been given intraoperative steroids during their biopsy were categorized in the nonsteroid group. Patients were excluded if they had PCNSL or relapsed or refractory DLBCL.

### Statistical Analysis

Statistical analyses were performed using Excel version 365 (Microsoft), Stata MP version 18 (StataCorp), and R version 4.5.1 (R Project for Statistical Computing). Categorical variables were compared using χ^2^ tests or Fisher exact tests, while continuous variables were assessed with unpaired *t* tests, as appropriate. Modified Poisson regression models with robust SEs were applied to identify factors associated with diagnostic first biopsies and estimate prevalence ratios (PRs). For the steroid-treated group, odds ratios (ORs) for diagnostic biopsy were estimated by Firth penalized logistic regression due to sparse data. Statistical significance was defined as a *P* value less than .05. Missing data were excluded from analyses.

## Results

### Demographic and Clinical Characteristics

A total of 320 patients with DLBCL at our institution met the inclusion criteria (Figure). Of the patients, 164 (51%) were female, and the mean (SD [range]) age was 68 (14 [17-96]) years in both groups. Forty-eight (15%) received steroids prebiopsy, and 272 (85%) did not. The proportion of female patients was significantly lower in the group that received steroids prebiopsy (35% vs 54%; difference, −19 percentage points [pp]; 95% CI, −33 pp to −4 pp; *P* = .03). The majority of patients in both groups had stage IV disease at the time of diagnosis, and the mean stage was III in both groups. Other lymphoma characteristics, including mean LDH at time of diagnosis, proportion with double-hit status, and mean IPI score, were not significantly different between the 2 groups (Table 1).

**Figure.**
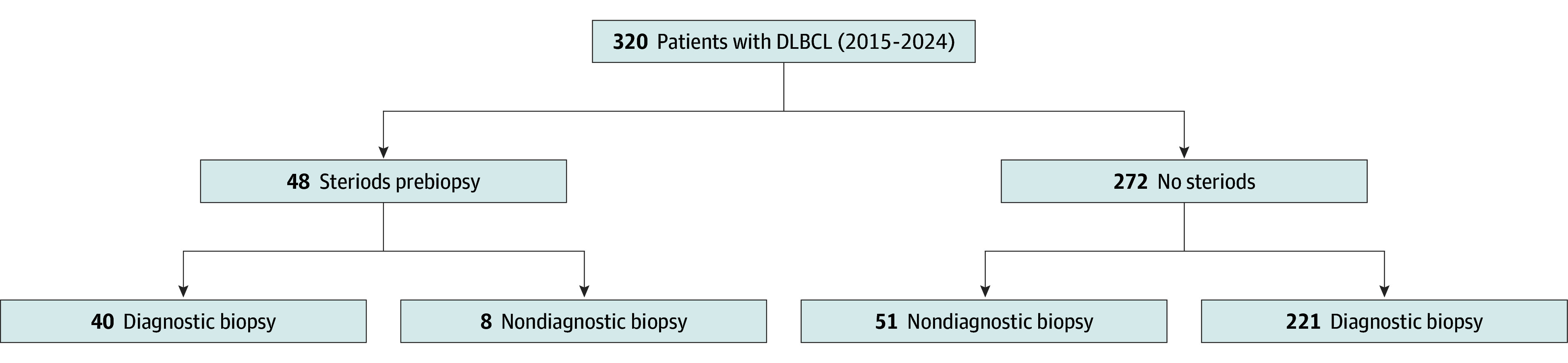
Study Population That Met Inclusion Criteria: Patients With Diffuse Large B-Cell Lymphoma (DLBCL) Diagnosed at Tertiary Hospitals Patients were stratified by administration of steroids before their biopsy and evaluated for diagnostic biopsies.

**Table 1.** Description of the Study Population in 2 Categories: Steroids Before Biopsy and Steroid-Naive Before Biopsy

Characteristics	Individuals, No. (%)		*P* value, univariate
	Steroids (n = 48)	Nonsteroids (n = 272)	
Age, mean (SD), y	67 (14)	68 (14)	.53
Sex			
Female	17 (35)	147 (54)	.02
Male	31 (65)	125 (46)	.02
Stage			
I	10 (21)	39 (14)	NA
II	6 (12)	38 (14)	
III	5 (10)	52 (19)	
IV	27 (56)	143 (53)	
Mean stage (SD)	3.02 (1.25)	3.10 (1.11)	.68
LDH, mean (SD), U/L	422 (378)	368 (413)	.40
Double-hit status	8 (17)	26 (10)	.22
IPI score			
0-1	3 (12)	23 (16)	NA
2-3	15 (58)	70 (50)	
4-5	8 (31)	48 (34)	
Mean IPI score (SD)	2.92 (1.26)	2.84 (1.29)	.75
First biopsy positive	40 (83)	221 (81)	.89
Repeat biopsy positive	7 (88)	49 (96)	.50
Type of biopsy			
Excisional/incisional	11 (23)	84 (31)	.42
Core needle	30 (63)	134 (49)	
Fine needle aspiration	2 (4)	29 (11)	
Endobronchial biopsy	1 (2)	5 (2)	
Endoscopic biopsy	2 (4)	16 (6)	
Other	2 (4)	4 (1)	
Days from first negative biopsy to treatment, mean (SD)	40 (39)	51 (28)	.46
Reasons for negative biopsy			
Insufficient material	5 (56)	32 (63)	.52
Necrosis and apoptosis	3 (33)	9 (18)	
Crush artifact	1 (11)	10 (20)	

Abbreviations: IPI, International Prognostic Index; LDH, lactate dehydrogenase; NA, not applicable.

SI conversion factor: To convert LDH to μkat/L, multiply by 0.0167.

### Steroid Treatment, Other Characteristics, and Association With Diagnosis

Within the steroid group, 40 out of 48 (83%) first biopsies were diagnostic of DLBCL, and in the nonsteroid group, 221 out of 272 (81%) first biopsies were diagnostic; the difference was not statistically significant (PR, 1.03; 95% CI, 0.89-1.18; *P* = .72) (Table 2). Adjusting for age, gender, and type of biopsy did not alter the results (PR, 0.99; 95% CI, 0.88-1.12; *P* = .92). The majority of patients (164 of 320 [51%]) underwent a core needle biopsy, followed by excisional or incisional (95 of 320 [30%]), and fine needle aspiration (31 of 320 [10%]) (Table 1). Only 9 fine needle aspiration biopsies (29%) were diagnostic compared with 137 core needle biopsies (84%), 90 excisional or incisional biopsies (95%), and 5 other biopsy types (83%) (*P* < .001) (Table 2). Compared with excisional or incisional biopsies, core needle and fine needle aspiration were 12% (PR, 0.88; 95% CI, 0.81-0.96; *P* = .01) and 69% (PR, 0.31; 95% CI, 0.18-0.53; *P* < .001) less likely to be diagnostic, respectively.

**Table 2.** Characteristics by Diagnostic First Biopsy and Prevalence Ratio (PR)

Characteristics	Individuals, No. (%)		*P* value	Individuals, No. (%)
	Diagnostic biopsy (n = 262)	Nondiagnostic biopsy (n = 59)		Unadjusted PR (95% CI)
Steroid use				
Yes	40 (83)	8 (17)	.73	1.03 (0.89-1.18)
No	221 (81)	51 (19)		1 [Reference]
Age, mean (SD), y	68 (14)	64 (15)	.05	1.00 (0.99-1.01)
Sex				
Female	129 (79)	35 (21)	.17	0.93 (0.84-1.03)
Male	132 (85)	24 (15)		1 [Reference]
Stage				
I	37 (76)	12 (24)	.44	1 [Reference]
II	38 (86)	6 (14)		1.14 (0.94-1.39)
III	49 (86)	8 (14)		1.14 (0.94-1.38)
IV	137 (81)	33 (19)		1.07 (0.90-1.27)
LDH (n = 301), mean (SD), U/L	383 (383)	342 (511)	.50	1.00 (0.99-1.00)
Double-hit status				
Yes	30 (88)	4 (12)	.29	1.09 (0.95-1.25)
No	231 (81)	55 (19)		1 [Reference]
Type of biopsy				
Excisional/incisional	90 (95)	5 (5)	<.001	1 [Reference]
Core needle	137 (84)	27 (16)		0.88 (0.81-0.96)
Fine needle aspiration	9 (29)	22 (71)		0.31 (0.18-0.53)
Endobronchial biopsy	3 (50)	3 (50)		0.53 (0.24-1.18)
Endoscopic biopsy	17 (94)	1 (6)		0.99 (0.88-1.13)
Other	5 (83)	1 (17)		0.88 (0.61-1.26)

Abbreviation: LDH, lactate dehydrogenase.

SI conversion factor: to convert LDH to μkat/L, multiply by 0.0167.

### Nondiagnostic Biopsy Characteristics

Among patients with a nondiagnostic first biopsy, repeat biopsies were successful in establishing a diagnosis in 7 of 8 patients (88%) in the steroid group and 49 of 51 patients (96%) in the nonsteroid group; this difference was not statistically significant (PR, 0.91; 95% CI, 0.70-1.19). The mean (SD) time from the first nondiagnostic biopsy to treatment initiation was 40 (39) days in the steroid group and 51 (28) days in the nonsteroid group, with no statistically significant difference between the groups.

Additionally, there was no difference between histological findings on the biopsy for those who received steroids compared with those who did not receive steroids. The most common reason for both patient populations for having a nondiagnostic biopsy was insufficient material (5 patients [56%] in the steroid group vs 32 [63%] in the nonsteroid group). This was followed by necrosis and apoptosis in patients who received steroids (3 patients [33%]) and crush artifacts in those who did not receive steroids (1 patient [20%]) (Table 1).

### Steroid Treatment Characteristics

The most common reasons for steroid initiation were symptoms from mass effect and CNS or neurologic symptoms from secondary CNS involvement. Other reasons were varied: treatment of rash, facial swelling, symptomatic splenomegaly, and suspected infection (Table 3). The mean total dose of steroids was higher among patients who had an initial diagnostic biopsy compared with those who had a nondiagnostic biopsy, although the difference was not statistically significant (297 vs 103 mg in prednisone equivalents). Twenty out of 40 patients (50%) who had a diagnostic biopsy received dexamethasone, whereas 1 out of 8 patients (13%) who had a nondiagnostic biopsy received dexamethasone. The mean (SD) duration of steroid treatment was similar between those who had a diagnostic vs nondiagnostic biopsy (8 [10] days vs 9 [13] days), and approximately one-third of patients had steroid treatment withdrawn in the days to weeks before biopsy. Unadjusted odds ratios for steroid dose, duration, and withdrawal are in the eTable in Supplement 1. A statistically significant difference was observed in the types of biopsies performed between those who had a diagnostic biopsy and those with a nondiagnostic biopsy. A higher proportion of excisional and incisional biopsies were performed in the diagnostic biopsy group compared with the nondiagnostic biopsy group, with more fine needle aspiration performed in the nondiagnostic group (OR, 0.01; 95% CI, 0.001-0.55; *P* = .03).

**Table 3.** Cases of Diffuse Large B-Cell Lymphoma in Which Steroids That Were Administered Before Biopsy Were Further Categorized by Diagnostic and Nondiagnostic Biopsies^a^

Characteristic	Individuals, No. (%)		*P* value
	Diagnostic biopsy (n = 40)	Nondiagnostic biopsy (n = 8)	
Total dose of steroids in prednisone equivalents, mean (SD)	297 (326)	103 (37)	.10
Mean total days on steroids (SD)	8 (10)	9 (13)	.86
Type of steroid			
Dexamethasone	20 (50)	1 (13)	.05
Nondexamethasone	20 (50)	7 (88)	
Reason for steroid initiation			
CNS or neurologic symptoms	9 (23)	1 (13)	.93
Abdominal or GIT symptoms	1 (3)	0	
Symptoms from mass effect	9 (23)	2 (25)	
Respiratory symptoms	6 (15)	1 (13)	
Other	15 (38)	4 (50)	
Type of biopsy			
Excisional/incisional	11 (28)	0	.01
Core needle	25 (63)	5 (63)	
Fine needle aspiration	0 (0)	2 (25)	
Endobronchial biopsy	1 (3)	0	
Endoscopic biopsy	2 (5)	0	
Other	1 (3)	1 (13)	
Steroids withdrawn	14 (35)	3 (38)	.89
Days off steroids, mean (SD)	3 (7)	6 (11)	.44

Abbreviations: CNS, central nervous system; GIT, gastrointestinal tract.

^a^ Groups were analyzed by steroid characteristics, symptoms prompting steroid initiation, and type of biopsy.

#### Biopsy Site and Temporal Trends

Further analysis was conducted on the association of biopsy site (grouped into lymph node, extranodal mass, and bone marrow biopsy) and temporal trends with diagnostic yield. Diagnostic positivity was observed in 5 of 6 bone marrow biopsies (83.3%), 88 of 109 lymph node biopsies (80.7%), and 168 of 205 extranodal mass biopsies (82.0%). The differences were not statistically significant. Logistic regression models stratified by biopsy type—including excisional, incisional, core needle, FNA, endoscopic, and other biopsy methods—showed no statistically significant temporal trends in diagnostic yield across the study period. The odds ratios for diagnostic success per calendar year ranged from 0.91 to 1.55 across biopsy types, with all 95% CIs including 1.0 and *P* values greater than .05.

## Discussion

This single-institution retrospective study examined the association of prebiopsy corticosteroid administration and diagnostic yield of biopsies in patients with newly diagnosed DLBCL. Contrary to longstanding clinical caution, we found no significant difference in diagnostic yield between patients who received corticosteroids and those who did not. Additionally, no associations were observed between diagnostic biopsy rates and cumulative steroid dose, duration of steroid use, steroid type, or steroid withdrawal before biopsy. When the first biopsy was nondiagnostic, repeat biopsy rates remained high across both groups, and there was no significant difference in time to initiation of chemotherapy. These findings collectively suggest that steroid exposure does not delay clinically meaningful outcomes and challenge the prevailing notion that corticosteroid use compromises diagnostic accuracy. Instead, they support the view that corticosteroids may be administered when clinically indicated, without jeopardizing diagnostic efforts.

The limited body of evidence addressing prebiopsy steroid use in lymphoma largely centers on PCNSL and yields mixed conclusions. Earlier retrospective studies focusing on pediatric mediastinal lymphoma and PCNSL respectively, reported reduced diagnostic rates in steroid-treated groups.^9,15^ While one of these studies found no correlation between steroid dose or duration and diagnostic accuracy,^15^ another noted that steroid use beyond one week before biopsy was associated with reduced diagnostic yield.^9^ More recent retrospective studies, however, align more closely with our findings, demonstrating no difference in diagnostic accuracy between steroid-treated and untreated patients in PCNSL cohorts.^16,17,19^ One of these studies also found no association between steroid dose or duration and biopsy yield.^19^ A multicenter retrospective study reported that while steroid use was associated with an increased need for repeat biopsies, it did not significantly impact the overall diagnostic delay.^18^

Steroid withdrawal before biopsy has been explored as a strategy to mitigate diagnostic interference. Our findings revealed no association between steroid withdrawal and diagnostic yield, consistent with a previous retrospective cohort in PCNSL.^19^ Interestingly, one study found that steroid withdrawal did not reduce the risk of a nondiagnostic biopsy and was instead associated with increased diagnostic delays, suggesting potential drawbacks to withholding steroids in patients who may benefit symptomatically.^18^

Another area of uncertainty in the literature is the influence of steroid type on diagnostic performance. A case-control study examining patients with aggressive B-cell lymphomas reported that intravenous dexamethasone, particularly at high doses administered over shorter intervals, was associated with altered CD20 staining patterns.^10^ These changes, along with steroid-induced histiocytic infiltration and disruption of diffuse lymphoma cell patterns, contributed to diagnostic complexity. While we did not assess morphologic or immunohistochemical alterations in our case series, we found no significant difference in first biopsy diagnostic rates or treatment delays based on steroid type. The aforementioned case-control study also reported that extensive necrosis and apoptosis were similarly prevalent in both steroid-treated and untreated groups—findings that align with our results and reflect the inherently aggressive nature of DLBCL.

Notably, biopsy modality emerged as more greatly associated with diagnostic success than steroid exposure. Excisional and incisional biopsies had the highest diagnostic yields, followed by core needle biopsies and, lastly, fine needle aspiration. This finding reinforces well-established literature emphasizing the importance of obtaining sufficient tissue with preserved architecture for lymphoma diagnosis.^20,21^ Additionally, diagnostic yield was similar across biopsy sites and types, and no significant temporal trends were observed over the study period.

Lastly, a surprising and potentially significant finding was the lower proportion of women in the steroid-treated group. While this may partly reflect sample size limitations, it is consistent with broader trends in health care where female patients’ symptoms are under-recognized or under-treated, particularly in acute care settings.^22,23,24^ This discrepancy may contribute to differential clinical decision-making, with fewer women being prescribed corticosteroids despite comparable symptom severity. This gender disparity warrants further investigation and underscores the need for equitable approaches to symptom management and diagnostic support during lymphoma evaluation.

### Future Research

Future research should seek to validate and expand upon these findings through multi-institutional retrospective studies or ideally prospective designs. Including other aggressive lymphoma subtypes (eg, Burkitt or primary mediastinal B-cell lymphoma) would enhance generalizability especially in clinical situations where a presenting patient with findings of aggressive lymphoma do not have an established histologic diagnosis. Additionally, in resource-constrained settings where delays in biopsy are common, understanding whether corticosteroids can be safely administered without compromising diagnostic outcomes is of high clinical relevance. Such research could inform guidelines that support both effective symptom management and diagnostic accuracy in diverse health care environments.

### Limitations

Several limitations of our study must be acknowledged. As a retrospective analysis, it is limited by potential selection bias and reliance on documentation quality. Importantly, our case series included only patients with a confirmed DLBCL diagnosis, thereby excluding those who may have had lymphoma but were never diagnosed due to nondiagnostic biopsy—possibly influenced by steroid use. Additionally, our surrogate measures of disease burden may not fully capture the complexity of clinical presentation or patient symptoms. The sample size, while meaningful, may lack power for more granular analyses of steroid dose, timing, or type.

## Conclusions

In this case series of 320 patients with DLBCL, we found that prebiopsy corticosteroid administration was not significantly associated with reduced diagnostic yield in DLBCL nor time to treatment initiation. Biopsy technique was most strongly associated with diagnostic success. These findings support a more individualized and pragmatic approach to corticosteroid use, especially in symptomatic patients and in settings where logistical delays in diagnosis are common. Further prospective and inclusive research is essential to refine these conclusions and to guide balanced, evidence-based clinical decision-making.

## Notes

Supplement 1. eTable. Unadjusted Logistic Regression for Steroid Use and Diagnostic First Biopsy

Supplement 2. Data Sharing Statement
