# Supplementary material for: Prebiopsy Steroids and Diagnostic Yield in Patients With Diffuse Large B-Cell Lymphoma
Source: JAMA Netw Open. 2025 Dec 11;8(12):e2548617. doi: 10.1001/jamanetworkopen.2025.48617 (PMC12699353; doi:10.1001/jamanetworkopen.2025.48617)
Supplement: Supplement 1. — eTable. Unadjusted Logistic Regression for Steroid Use and Diagnostic First Biopsy [file jamanetwopen-e2548617-s001.pdf]

Supplemental Online Content

Madireddy S, Pou S, Mandi K. Prebiopsy Steroids and Diagnostic Yield in Diffuse Large B-Cell Lymphoma. *JAMA Netw Open*. 2025;8(12):e2548617.
doi:10.1001/jamanetworkopen.2025.48617

eTable. Unadjusted Logistic Regression for Steroid Use and Diagnostic First Biopsy

This supplemental material has been provided by the authors to give readers additional information about their work.

© 2025 Madireddy S et al. *JAMA Network Open*.

eTable. Unadjusted Logistic Regression for Steroid Use and Diagnostic First Biopsy

Characteristics	OR (95% CI)	p-value
**Total days of steroids (per day)**	0.99 (0.93-1.06)	0.75
**Steroids withdrawn (vs. maintained)**	0.86 (0.19-3.80)	0.84
**Total dose of steroids (per mg)**	1.01 (0.99-1.02)	0.16

Abbreviations: OR, odds ratio; CI, confidence interval.
Firth’s penalized logistic regression was applied due to the limited number of outcomes.

© 2025 Madireddy S et al. *JAMA Network Open*.
