# Supplementary material for: Prebiopsy Steroids and Diagnostic Yield in Patients With Diffuse Large B-Cell Lymphoma
Source: JAMA Netw Open. 2025 Dec 11;8(12):e2548617. doi: 10.1001/jamanetworkopen.2025.48617 (PMC12699353; doi:10.1001/jamanetworkopen.2025.48617)
Supplement: Supplement 2. — Data Sharing Statement [file jamanetwopen-e2548617-s002.pdf]

Data Sharing Statement

Madireddy. Prebiopsy Steroids and Diagnostic Yield in Diffuse Large B-Cell Lymphoma. *JAMA Netw Open*. Published December 11, 2025. doi:10.1001/jamanetworkopen.2025.48617

Data
Data available: Yes
Data types: Deidentified participant data
How to access data: smadireddy@brownhealth.org
When available: With publication

Supporting Documents
Document types: Statistical/analytic code
How to access documents: smadireddy@brownhealth.org
When available: With publication

Additional Information
Who can access the data: researchers whose proposed use of the data has been approved
Types of analyses: chi-square, fishers test, logistic regression
Mechanisms of data availability: after approval of a proposal, and with a signed data access agreement
